# Multiple myeloma in a man with breast cancer: A case report and literature review

**DOI:** 10.1097/MD.0000000000046540

**Published:** 2025-12-19

**Authors:** Xin Zhou, Meng-Ran Li, Xiao-Hui Sui, Ning-ning Shan

**Affiliations:** a Department of Hematology, Shandong Provincial Hospital Affiliated to Shandong First Medical University, Jinan, Shandong Province, China; b Department of Hematology, The Second Hospital of Shandong University, Jinan, Shandong Province, China.

**Keywords:** biopsy, breast cancer, extramedullary metastasis, multiple myeloma

## Abstract

**Rationale:**

The development of multiple myeloma (MM) following male breast cancer is extremely rare and can often be mistaken for bone metastases in the early stages, potentially leading to delays in diagnosis and treatment.

**Patient concerns:**

In this case report, we describe a 68-year-old male patient who presented with multiple osteolytic lesions on imaging following breast cancer surgery, and was ultimately diagnosed with MM via bone marrow biopsy.

**Diagnoses:**

MM.

**Interventions:**

After being diagnosed with MM, the patient was treated with various chemotherapy regimens, with the subsequent emergence of rare extramedullary disease necessitating an adjustment in therapy.

**Outcomes:**

The patient successfully underwent autologous hematopoietic stem cell transplantation but later succumbed to a novel coronavirus infection.

**Lessons:**

This case highlights the diagnostic challenge of misattributing osteolytic lesions to breast cancer and underscores the importance of biopsy in patients with metastatic disease.

## 1. Introduction

Multiple myeloma (MM), as a common hematologic malignancy, presents diagnostic challenges in many patients.^[1]^ Breast cancer is the most prevalent cancer among women and is a serious disease requiring treatment.^[2]^ Male breast cancer, however, has a much lower incidence, accounting for 0.6% of global breast cancer cases, with a standardized incidence rate of 0.4 per 100,000, and remains stable at around 35 per 100,000 in developing countries.^[3]^ Following surgical treatment, patients often require adjuvant chemotherapy or chemoradiotherapy. These treatments are frequently associated with hematologic toxicity.^[4]^ Oncologists typically interpret changes in blood test indicators as treatment side effects and rarely consider the possibility of a concurrent tumor, particularly a hematologic malignancy. The proportion of MM patients presenting with breast cancer as a synchronous or metachronous cancer was 1.6%.^[5,6]^ Few reports have described cases of male breast cancer with MM.^[7]^ The following text presents a case report of a male patient who developed MM after breast cancer.

## 2. Case presentation

A 68-year-old man was diagnosed with stage I (pT1N0M0) infiltrating ductal carcinoma in September 2016. The tumor was estrogen receptor and progesterone receptor positive, with a Ki-67 index of 30%. FISH analysis revealed HER2/CEP17 ratios of 1.4 and 1.47 in 2 samples, both below the 2.0 diagnostic threshold, confirming HER2-equivocal status. Initial treatment consisted of the TAC regimen and letrozole.

Two years post-therapy, the patient developed bone pain. Bone ECT showed multiple osteolytic lesions in the ribs and ischium, indicating metastatic disease. A bone biopsy was recommended but refused. In male breast cancer patients, bone lesions appearing 2 years after chemotherapy primarily suggest breast cancer metastasis. Thus, letrozole plus everolimus (125 mg, qd) was initiated. However, severe oral mucositis led to switching to anastrozole plus neratinib for 5 months, with no significant pain improvement. In June 2020, repeat ECT revealed widespread bone metastasis. GX regimen (gemcitabine + capecitabine) was administered. By October, right shoulder pain prompted an MRI, showing multiple enhanced lesions in the right humerus, clavicle, and scapula, consistent with metastasis. Due to disease progression, treatment was adjusted to paclitaxel (250mg, d1,8) plus anlotinib (8mg, po, d1–14), with denosumab (120mg, q4w) for bone protection. In March 2021, chest and abdominal computed tomography (CT) confirmed progressive bone metastases. Therapy was changed to fulvestrant (0.5g) plus everolimus (75mg, d1–21, q4w) with adjunct radiotherapy. Despite multiple chemotherapy and endocrine adjustments, bone pain worsened and osteolytic lesions expanded, raising suspicion of another underlying disease.

**Figure 1.**
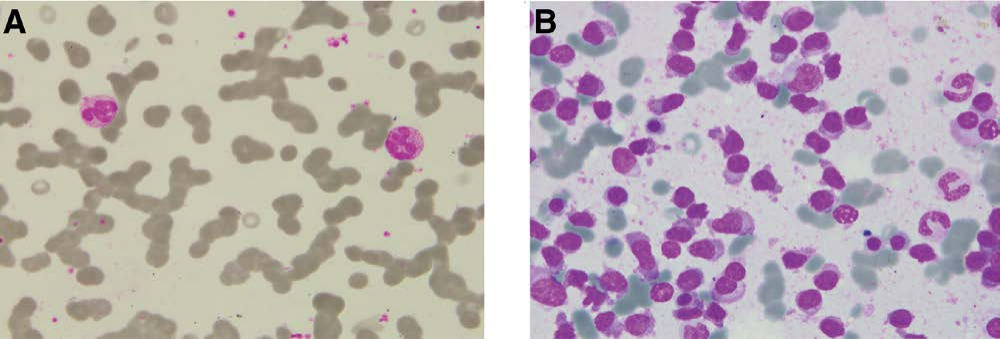
Smear results. (A) The peripheral blood smear reveals a large number of coin-shaped cells. (B) The bone marrow smear reveals a large number of plasma cells.

In May 2021, the patient presented with fatigue, nausea, and vomiting. Fulvestrant was initially suspected and discontinued. Further evaluation revealed renal failure (creatinine 274.35 µmol/L) (Reference ranges:40–135µmol/L) and peripheral blood smear showing numerous coin-shaped cells (Fig. 1A). Serum protein electrophoresis detected monoclonal gammopathy: IgG 42.40 g/L (Reference ranges:7–16 g/L), free kappa light chains 11,900 mg/L (Reference ranges:6.7–22.4 mg/L), and immunofixation confirmed IgG-kappa positivity, with hyperkalemia (6.30 mmol/L) (Reference ranges:3.5–5.5 mmol/L) and normocalcemia. MM was diagnosed, though no bone biopsy was performed. Bone marrow examination (Fig. 1B) and flow cytometry supported the diagnosis, showing 0.15% monoclonal plasma cells positive for CD138, CD38, CD28, and kappa. FISH detected IgH rearrangement. PET-CT confirmed the findings. Staged as ISS III, the patient started VDD regimen, with improved renal function after 1 cycle (creatinine 169 µmol/L). Due to neuropathy, treatment was switched to 4 cycles of IDD. By October 2021, symptoms recurred with IgG 16.10 g/L (Reference ranges:7–16 g/L), kappa light chains 2200 mg/L (Reference ranges:6.7–22.4 mg/L), and 40% plasma cell infiltration in bone marrow. Flow cytometry showed 9.5% abnormal plasma cells (CD138+, CD38+, cKappa+, partial CD56+, CD19–). In November, treatment was changed to DRD, resulting in symptom relief and partial response.

**Figure 2.**
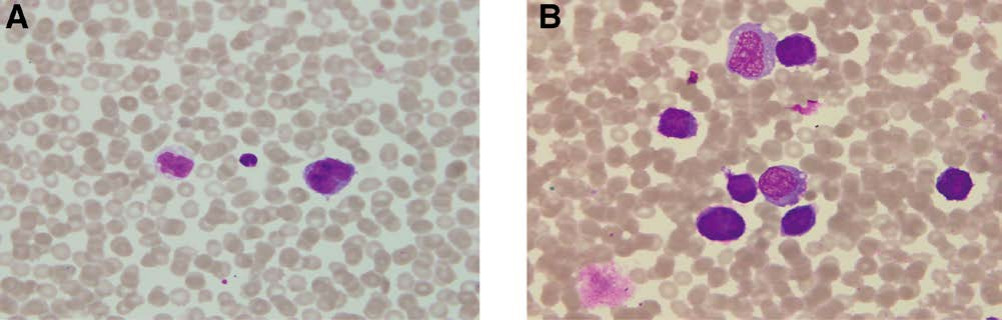
The result of exfoliative cytology examination. Malignant tumor cells are observed in the (A) pericardial effusion and (B) pleural effusion.

In April 2022, the patient was readmitted with intermittent fatigue, chest tightness, and dyspnea. Cardiac ultrasound identified large pericardial effusion with restricted ventricular diastolic function and mild bilateral pleural effusion. Pericardiocentesis was performed, draining 800 to 1000 mL of hemorrhagic fluid daily, with symptomatic improvement. Cytology of the effusion revealed malignant cells (Fig. 2A and B). Flow cytometry showed CD38 negativity; given the prior daratumumab exposure, myeloma cell infiltration was suspected. Treatment was switched to the KBD regimen. After the first carfilzomib dose, dyspnea alleviated and pericardial effusion markedly decreased. Four KBD cycles resulted in very good partial response (VGPR). The patient subsequently underwent successful stem cell collection and autologous hematopoietic stem cell transplantation with engraftment. Unfortunately, the patient later died of a novel coronavirus infection. (The treatment timeline is shown in Table 1.)

**Table 1:** Clinical treatment timeline.

Point of time	Clinical events	Examination/treatment
September 2016	Breast cancer diagnosis	TAC regimen (Cyclophosphamide 1g, Docetaxel 140 mg, Doxorubicin 140 mg q3w × 6)
September 2019	Bone pain	ECT revealed multiple osteolytic lesions/ letrozole and everolimus (125 mg, qd).
June 2020	-	Repeat bone ECT showed widespread bone metastasis/GX regimen (Gemcitabine + Capecitabine).
March 2021	-	Enhanced CT scans showed multiple bone metastases/fulvestrant (0.5 g) combined with everolimus (75 mg, po, d1–21, q4w)
May 2021	MM diagnosis	VDD regimen (liposomal Doxorubicin 60 mg dL, Bortezomib 2.4 mg dL, 4, 8, 11, Dexamethasone 20 mg dL, 2, 4, 5, 8, 9, 11, 12)
November 2021	Fatigue and bone pain	DRD (Lenalidomide 25 mg qod d1–21, Daratumumab 500 mg d1–2, 1000 mg d15, Dexamethasone 20 mg dL, 2, 8, 9, 15, 16, 21, 22).
April 2022	Rare extramedullary metastasis	KBD (Carfilzomib 30 mg dL, 2, 60 mg d8, 9, 15, 16; Bendamustine 125 mg dL, 2; Dexamethasone 20 mg dL, 2, 8, 9, 15, 16, 22, 23)
November 2022	ASCT	The total number of mononuclear cells (MNC) and CD34+ cells infused was 6.82 × 10⁸/L and 4.29 × 10⁶/L.

ASCT = autologous stem cell transplantation, ECT = emission computed tomography.

## 3. Discussion

Secondary malignancies in cancer survivors are an emerging concern in oncology. Among a cohort of 15,398 malignant tumor cases, the proportion of breast cancer patients who developed a second primary cancer was 1.22%,^[8]^ the proportion of MM patients presenting with breast cancer as a synchronous or metachronous cancer was 1.6%.^[5,6]^ Current literature lacks large-scale studies, with most evidence being documented in isolated case reports. Dorffel et al.^[9]^found an increased incidence of MM in patients diagnosed with breast cancer, while Cao et al.^[10]^observed extramedullary infiltration of MM in post-surgical breast cancer samples, which corroborates our case. Trisal et al.^[11]^reported 2 cases of hypercalcemia in patients with metastatic breast cancer and MM, which contrasts with our case, as calcium levels remained normal in our patient, a fact that could have delayed the final diagnosis.

Epidemiological studies have confirmed that breast cancer survivors have an increased risk of developing a second malignancy due to the genotoxic effects of chemotherapy, radiotherapy, and hormone therapy.^[12–14]^ However, recent studies have revealed a paradoxical decline in MM incidence among breast cancer patients who received radiotherapy.^[15]^ Male breast cancer (MBC) itself accounts for only 1% of all breast cancers, and cases involving subsequent MM are exceedingly rare. Cornelly et al^[7]^ reported a case of a 74-year-old male breast cancer patient in whom CD138⁺ plasma cells were detected within the breast mass upon pathological examination, ultimately leading to a diagnosis of metastatic MM to the breast via bone marrow biopsy. It is worth noting that the risk profile for secondary malignancies in male breast cancer patients may differ. Approximately 30% of male breast cancer patients carry BRCA2 germline mutations, which could contribute to genomic instability and promote malignant transformation of plasma cells.^[16,17]^ Furthermore, studies have demonstrated that bone marrow stromal cells secrete cytokines such as interleukin-6 (IL-6) and vascular endothelial growth factor (VEGF), which promote both bone metastasis of breast cancer cells and clonal proliferation of plasma cells.^[18]^ Although genetic testing was not performed in our case, the occurrence of dual malignancies may partially be attributed to this genetic mechanism driving a multi-step carcinogenic process.

When a breast cancer patient presents with osteolytic bone lesions, imaging studies may suggest the presence of metastatic lesions. In breast cancer, approximately 82.2% of bone metastases are osteolytic, typically exhibiting irregular or moth-eaten margins, while osteoblastic or mixed patterns are also observed, with the former more common in HER2-negative or poorly differentiated tumors.^[19]^ CT reveals osteolytic lesions with soft tissue infiltration or osteoblastic lesions with increased density, whereas magnetic resonance imaging (MRI) is highly sensitive for early bone marrow involvement. In contrast, MM primarily presents as multiple, well-defined “punched-out” lytic lesions, rarely with sclerosis. CT shows lytic defects without sclerotic rims, and MRI detects early diffuse or focal marrow infiltration,^[20]^ though neither modality can definitively establish the pathological diagnosis. Therefore, tissue biopsy remains the gold standard in such cases.

International guidelines (NCCN/ESMO) clearly state that for asymmetrical distribution, isolated lesions, or bone lesions with progression after treatment, image-guided biopsy should be performed early, with diagnostic accuracy reaching 85% and 95%.^[21,22]^ Given the lack of clear guidelines specifying which patients and which tumors require biopsy to confirm metastasis, each metastatic diagnosis should be carefully evaluated for its likelihood of being related to the primary tumor. Clayer and Duncan^[23]^ followed up 50 cancer patients who developed new bone lesions and found that 9 (15%) of the new bone lesions were unrelated to the primary tumor. Hamaoka et al^[24]^ further found that ~12% of breast cancer patients with bone metastasis were found to have a second primary tumor upon biopsy, and 9% of them had their treatment plan adjusted accordingly. Similarly, Simmons et al^[25]^ reported that 20% of metastatic breast cancer patients had metastatic lesions with a discordant hormone receptor or HER2 status from the primary tumor, and 38% of patients adjusted their treatment strategy. In this case, all the abnormal findings were initially considered as metastatic or recurrent breast cancer, but after shifting the treatment plan from standard breast cancer therapy to MM treatment, the patient showed clinical improvement. Studies indicate that 2% to 5% of MM patients have a history of solid tumors, and early biopsy can prevent misdiagnosis of symptoms as metastatic breast cancer.^[26]^ Unfortunately, the delay in MM diagnosis and treatment occurred due to the failure to perform timely bone biopsy.

In this case, the patient developed rare extramedullary disease (EMD) involving the pericardium and pleura. At initial diagnosis, EMD occurrence is ~4.8% to 7%, while progression or relapse-associated EMD accounts for ~6% of cases. Pericardial and pleural metastases represent exceptionally rare subtypes of EMD, which typically signify aggressive tumor biology.^[27]^ A pooled case analysis spanning 1970 to 2002 identified only 6 reported instances of MM-associated pericardial effusion, with direct plasmacytic infiltration of the pericardium being even rarer.^[28]^ Furthermore, among 3480 cases of pleural effusion examined in a separate study, merely 2 (0.06%) were attributed to MM.^[29]^ In this case, carfilzomib demonstrated promising efficacy. Furthermore, several emerging approaches also offer new perspectives for cancer treatment.^[30–32]^ Notably, daratumumab induces internalization or downregulation of the CD38 antigen on tumor cells, leading to a false-negative result in flow cytometry.^[33]^

## 4. Conclusion

This case illustrates the diagnostic complexity of MM emerging after breast cancer, including rare extramedullary involvement. Timely biopsy of osteolytic or atypical lesions is essential for accurate diagnosis, particularly when clinical features overlap. Coexisting malignancies may delay diagnosis, necessitating a high index of suspicion for secondary tumors. Multidisciplinary evaluation and dynamic lesion assessment are crucial to avoid misdiagnosis and guide personalized therapy. When biopsy is unfeasible, thorough differential diagnosis remains vital. Future studies should investigate the mechanisms of dual tumorigenesis in breast cancer and MM, explore genetic and treatment-related interactions, and advance early detection through improved diagnostic tools.

## Author contributions

**Writing – original draft:** Xin Zhou, Meng-Ran Li.

**Writing – review & editing:** Xiao-Hui Sui, Ning-ning Shan.

## References

[1] Kazandjian D. Multiple myeloma epidemiology and survival: a unique malignancy. Semin Oncol. 2016;43:676–81. 28061985 10.1053/j.seminoncol.2016.11.004 PMC5283695

[2] Naik AM Joseph K Harris M Davis C Shapiro R Hiotis KL. Indigent breast cancer patients among all racial and ethnic groups present with more advanced disease compared with nationally reported data. Am J Surg. 2003;186:400–3. 14553859 10.1016/s0002-9610(03)00282-4

[3] Liu S Tang Y Li J Zhao W. Global, regional, and national trends in the burden of breast cancer among individuals aged 70 years and older from 1990 to 2021: an analysis based on the global burden of disease study 2021. Arch Public Health. 2024;82:170. 39343976 10.1186/s13690-024-01404-3 PMC11440909

[4] Marinopoulos S Skorda L Karatapanis S Rasidakis A. Multiple myeloma emerging after chemotherapy for non-small-cell lung cancer. Med Oncol. 2008;25:415–8. 18345519 10.1007/s12032-008-9056-0

[5] Turgutkaya A Yavaşoğlu I Şahin T Sargin G Bolaman AZ. Multiple myeloma and frequency of synchronous and second primary malignancies. J Hematopathol. 2021;14:197–203.

[6] Gulmez A. Breast cancer after multiple myeloma treatment. Curr Probl Cancer. 2019;43:100463. 30738577 10.1016/j.currproblcancer.2019.01.004

[7] Cornelly A Anas F. About a particular breast tumor of a man: a case report. Int J Adv Res. 2018;6:189–92.

[8] Liu Z Liu C Guo W Li S Bai O. Clinical analysis of 152 cases of multiple primary malignant tumors in 15,398 patients with malignant tumors. PLoS One. 2015;10:e0125754. 25945938 10.1371/journal.pone.0125754 PMC4422700

[9] Dörffel WV Reitzig P Dörffel Y Possinger K. Secondary malignant neoplasms in patients with breast cancer. Zentralbl Gynakol. 2000;122:419–27. 11005133 10.1055/s-2000-10604

[10] Cao S Kang HG Liu YX Ren X-B. Synchronous infiltrating ductal carcinoma and primary extramedullary plasmacytoma of the breast. World J Surg Oncol. 2009;7:43. 19393076 10.1186/1477-7819-7-43 PMC2680856

[11] Trisal D Kumar N Sundriyal D Gadpayle AK. Hypercalcaemia of malignancy: two primaries in the same patient. BMJ Case Rep. 2014;2014:bcr2014204368. 10.1136/bcr-2014-204368 PMC4069703 24962487

[12] Morton LM Onel K Curtis RE Hungate EA Armstrong GT. The rising incidence of second cancers: patterns of occurrence and identification of risk factors for children and adults. Am Soc Clin Oncol Educ Book. 2014;34:e57–67. 10.14694/EdBook_AM.2014.34.e57 24857148

[13] Henderson TO Moskowitz CS Chou JF. Breast cancer risk in childhood cancer survivors without a history of chest radiotherapy: a report from the childhood cancer survivor study. J Clin Oncol. 2016;34:910–8. 26700127 10.1200/JCO.2015.62.3314 PMC4871997

[14] Paterson F Stanway S Gothard L. Second Primary Neoplasms Following a Diagnosis of Breast Cancer[G]// Ring A., Parton M. Breast Cancer Survivorship: Consequences of Early Breast Cancer and Its Treatment. Springer International Publishing; 2016:213–34.

[15] Hou N Wang Z Ling Y. Radiotherapy and increased risk of second primary cancers in breast cancer survivors: an epidemiological and large cohort study. Breast (Edinburgh, Scotland). 2024;78:103824. 39442313 10.1016/j.breast.2024.103824 PMC11532779

[16] Cipak L Watanabe N Bessho T. The role of BRCA2 in replication-coupled DNA interstrand cross-link repair in vitro. Nat Struct Mol Biol. 2006;13:729–33. 16845393 10.1038/nsmb1120

[17] Silvestri V Leslie G Barnes DR. Characterization of the cancer spectrum in men with germline BRCA1 and BRCA2 pathogenic variants: results from the consortium of investigators of modifiers of BRCA1/2 (CIMBA). JAMA Oncology. 2020;6:1218–30. 32614418 10.1001/jamaoncol.2020.2134 PMC7333177

[18] Haider MT Ridlmaier N Smit DJ Taipaleenmäki H. Interleukins as mediators of the tumor cell-bone cell crosstalk during the initiation of breast cancer bone metastasis. Int J Mol Sci. 2021;22:2898. 33809315 10.3390/ijms22062898 PMC7999500

[19] Bannoura S Nahouli H Noubani A Flaifel A Khalifeh I. Characteristics of breast cancer metastasizing to bone in a mediterranean population. Cureus. 2020;12:e11679. 33391916 10.7759/cureus.11679 PMC7769727

[20] Ha JY Jeon KN Bae K Choi BH. Effect of bone reading CT software on radiologist performance in detecting bone metastases from breast cancer. Br J Radiol. 2017;90:20160809. 28256905 10.1259/bjr.20160809 PMC5605069

[21] Gradishar WJ Moran MS Abraham J. Breast cancer, version 3.2022, NCCN clinical practice guidelines in oncology. J Natl Compr Cancer Netw. 2022;20:691–722. 10.6004/jnccn.2022.0030 35714673

[22] Coleman RE Croucher PI Padhani AR. Bone metastases. Nat Rev Dis Primers. 2020;6:83. 33060614 10.1038/s41572-020-00216-3

[23] Clayer M Duncan W. Importance of biopsy of new bone lesions in patients with previous carcinoma. Clin Orthop Relat Res. 2006;451:208–11. 16801861 10.1097/01.blo.0000229296.52216.77

[24] Hamaoka T Madewell JE Podoloff DA Hortobagyi GN Ueno NT. Bone Imaging in metastatic breast cancer. J Clin Oncol. 2004;22:2942–53. 15254062 10.1200/JCO.2004.08.181

[25] Simmons C Miller N Geddie W. Does confirmatory tumor biopsy alter the management of breast cancer patients with distant metastases? Ann Oncol. 2009;20:1499–504. 19299408 10.1093/annonc/mdp028 PMC2731014

[26] Edahiro T Ureshino H Yoshida T Fukushima N Ichinohe T. Challenging diagnosis of lytic bone lesions between multiple myeloma and bone metastasis of primary breast cancer. Cureus. 2023;15:e48880. 38111446 10.7759/cureus.48880 PMC10726101

[27] Usmani SZ Heuck C Mitchell A. Extramedullary disease portends poor prognosis in multiple myeloma and is over-represented in high-risk disease even in the era of novel agents. Haematologica. 2012;97:1761–7. 22689675 10.3324/haematol.2012.065698 PMC3487453

[28] Chang JH Chang SH Huh JW. Pericardial amyloidosis associated with light-chain myeloma. Korean J Med. 2013;84:755–8.

[29] Wang Z Xia G Lan L. Pleural effusion in multiple myeloma. Intern Med. 2016;55:339–45. 26875957 10.2169/internalmedicine.55.4733

[30] Deng Q Yang X Li Z. Hyperbaric oxygen: a multifaceted approach in cancer therapy. Med Gas Res. 2024;14:130–2. 40232688 10.4103/mgr.MEDGASRES-D-23-00028 PMC466986

[31] Wu J Wu GL Yang Q. Illuminating the future: NIR-II visualizes gas therapy for precision cancer treatment. Med Gas Res. 2024;14:172–4. 40434382 10.4103/mgr.MEDGASRES-D-23-00060 PMC11257177

[32] Binson VA Thomas S Subramoniam M. Non-invasive detection of early-stage lung cancer through exhaled breath volatile organic compound analysis. Med Gas Res. 2025;15:198–9. 40070188 10.4103/mgr.MEDGASRES-D-24-00101 PMC11918466

[33] Nijhof IS Casneuf T Van Velzen J. CD38 expression and complement inhibitors affect response and resistance to daratumumab therapy in myeloma. Blood. 2016;128:959–70. 27307294 10.1182/blood-2016-03-703439

